# Cheap Artificial Teeth, and Cheap Dental Operations in England

**Published:** 1851-10

**Authors:** 


### ARTICLE IX.

### *Cheap Artificial Teeth, and Cheap Dental Operations in England.*

In my last communication I instituted a comparison between the cheap advertising teeth-mongers and those more respectable members of the profession, who practice in a more private manner, and trust rather to reputation than to chicanery and humbug for success. And I think I succeeded in showing, very distinctly, that the public were cheated by either the one party or the other.

I shall now proceed to redeem my promise of exposing some of the tricks of the former class—both as to artificial work and the operation of plugging or plastering, and to effect this the more readily, I will introduce my readers to one of the class, (as a specimen of the whole.) A Mons. Le Humbug, a well known member of the two and six-penny fraternity, and a gentleman who has obtained an unenviable notoriety.

This Mons. Le Humbug advertises to stop a single tooth, permanently, with his indestructible, incompressible and indescribable cement, at the low price of 2*s*. 6*d*. each, and presuming that this wonderful cement is nothing more than a mixture of silver filings, quicksilver and bismuth, I beg to introduce him to the reader.

You must fancy Mons. Le Humbug bedecked in his gorgeous chintz flowered dressing gown, his head surmounted with a tinselled skull cap, fuming away a real Havana, and occasionally moistening his lips from a Dresden cup of chocolate or coffee—his eyes resting with intense delight on the newspaper in the interesting occupation of spelling his own name and tracing the efforts of his lubricous fancy in the form of an advertisement. As the poet says, however, time flies—it is 10 o'clock, and his pleasing occupations are disturbed by the more pleasing intimation that a pigeon is trapped and waiting in his sanctum to be artistically plucked. After a proper interval the coffee and segar are discarded, and the professor enters his studio with an air somewhat between a theatrical braggadocio and a veritable barber. After the usual allowance of good mornings, and some sapient observations on the weather, the following dialogue commences :

*Patient*—I perceive by your advertisement Mons. Le Humbug that you stop teeth at 2*s.* 6*d.* each.

*Professor*—Yaas saar.

*Patient*—As I have one that requires stopping, will you be kind enough to do it for me?

*Professor*—Ya-as sa-ar, will you be so kind ash to open your jaws—ah, this is the tooth, ah—are in a dreadful state, but I will stop it with my beautiful shement which will last your life time sa-ah, and longer than that.

And he immediately proceeds to plaster in his beautiful cement—holes produced by caries, indentations on the teeth, and the interstices between them, it matters not, all are plastered up with the beautiful shement—and having in a workmanlike manner levelled the surfaces, he without hesitation or consultation proceeds to beautify (i. e. destroy) the teeth by the application of his *mineral acid.* During the ten or fifteen minutes that this takes, the patient sets patiently in his chair, his mouth open with wonder as well as for the convenience of the operator, contemplating the proceedings of the good Samaritan with a kind of instinctive dread of the result. At length the professor announces the termination of his labors, and with great self-complacency, points out the benefits he has conferred on the patient's mouth, and explaining, in his own elegant phraseology, the great results that will accrue from his having stopped *ten* teeth and cleaned the whole, coolly demands ten guineas as his very moderate fee.

The poor patient is struck with horror and astonishment—God bless me Mons. Le Humbug, I had only *one* decayed tooth, and did not want any others stopped or cleaned, and I will not pay ten guineas, ten guineas really is too ridiculous. Let me tell you Mr. Professor, that I consider it as an attempt to swindle me out of my money. Well sa-are, replies the professor, pocketing the swindle—well sa-ar that is *my fee*, and I shall expect to be paid, or shall at once commence legal proceedings. After a few genteel compliments, and sundry hard words, too true to be bearable by any but the most unblushing swindler, the poor patient who begins to find himself in a fix with Mr. Extortioner, and endeavors to compromise the matter by an offer to pay part—indeed, he has not the whole amount in his pocket—but the worthy professor, who is not to be foiled in this way, kindly offers to take what ready cash he has, and his card and promise to pay the remainder. This arrangement being complete, he allows his victim to leave the house, and immediately gives instructions to one of those legal harpies, a low attorney, to proceed at once for the balance, or if his victim has shown the white feather, for the whole amount, *honestly* sinking the portion he has received. By the next post the *patient* receives a six and eight-penny billet, and being perchance rather nervous and not over anxious of appearing in a court of law, pays the demand, costs included, of course a mere trifle compared with the advantages he has received from Mons. Le Humbug, for in all human probability in less than a week or even in a few hours, the patient finds on examining his mouth he has swallowed (of course to the great benefit of his digestive organs) the whole of the professor's beautiful shement, while in a few weeks, he is convinced of the irreparable injury his beautifying acid has done to his teeth, and if per chance any portion of the cement remains, he is converted into a walking galvanic battery, and thus having been, by the seductive attractions of *cheap* dentism, fleeced in pocket and injured in person, he determines to employ a respectable non-advertising dentist, to repair, as much as may be, the damage occasioned by the two and six-penny practitioner.

Thus much of stopping; there is, however, another and more fertile source of swindling in the preparation of artificial teeth.

In these cases, as in the last, the patient has no sooner entered the precincts of dentism than the extortion and swindle commences. Having inquired the price of the different kinds of teeth, ticketed by advertisement-like remnants at a cutting shop, to commence from five shillings each. The *Professor* (and it must be remembered, that these kind of fellows profess a degree of shrewdness that would fit them for a very high station in the swell mob) measuring at a glance the pecuniary capabilities of his victim, and the probable amount of his brains, vary in his demand according to his indications. If he thinks him, and he is seldom out of his calculation, perfectly gullible, he will probably demand five pounds for a tooth, fabricated out of a piece of denture made and grooved whilst the patient is waiting; and this apology for a tooth being *forced* between two others and kept in that position by lateral pressure is said to be fixed by atmospheric pressure; or, in their own vulgar phraseology, by suction. Should the patient express a wish to have a tooth *on gold*, our Moses Humbug immediately warrants the superiority of his method above all others, and frequently succeeds in extorting three and occasionally five times the price that would be charged by a respectable dentist, for the finest materials and the most finished workmanship *properly* adapted to the mouth; for it must be kept in mind, that our worthy professor, when he is guilty of such extravagance at all, never uses gold of more than fourteen, or at the utmost sixteen carats. Indeed, since the discovery of the electrotype method of gilding, which he considers a godsend, he rarely ever uses this, but contents himself with gilded silver, brass or pinchback, which artificially colored and well polished, not only pass under the designation of gold, but are charged for as such.

The number of persons annually fleeced by these advertising gentry, is really wonderful, and not the less so that, generally speaking, they are of a rank in life that might be supposed to insure something like common sense and a knowledge of the world. Could any common sense person suppose, for instance, that a nobleman, and presumed to be a man of letters too—could put himself in the way of being victimised by one of these harpies, and yet such is the fact.

This nobleman was induced by some circumstance or other to drive to one of the fellows ; seeing him well dressed, a circumstance that has a marvellous effect in raising the price if not the quality of the article, demanded three guineas each for *his best* artificial substitutes.

The model was taken and the following day appointed for their insertion ; in the interim, however, Monsieur Jackall, who is tolerably well trained for the purpose, and frequently a partner in the proceeds, obtained the gentleman's name and address from his coachman.

At the time appointed on the day following, the nobleman was ushered into the presence of the professor, who immediately exclaimed, good morning, *my lord!* your lordship's teeth are ready, and very beautiful they are ; not made of the common material, *my lord*, such as I use for common people, *my lord*, but such as are peculiarly fitted for *your lordship's* mouth. Having thus lorded his patient, he inserted the tooth, and then a few more bows and scrapes. His lordship somewhat annoyed by all this fulsome nonsense, demanded the price, when he found *ten guineas* demanded for what he had agreed to give three. His lordship coolly put his hand to his mouth, removed the teeth, and presenting them to Mons. Le H., made his congee. A legal application was made, but his lordship not being made of spongeable material, was not to be frightened in that way. The case was eventually tried at Kingston, and a verdict returned in favor of his lordship, leaving to Monsieur the intense gratification of liquidating the legal expenses. The barefaced impudence of these fellows is, in some cases, so extraordinary, even for them, that it could scarcely be credited, unless coming from a source in which implicit confidence may be placed.

One of these tricks, and by no means an uncommon one, is called, making a set on spec, and consists in making a duplicate set; and when one set is in the mouth, presenting the other to the patient and demanding payment for both ; observing, he never makes one set alone in case of accident.

It might be thought, that such a barefaced swindle *could never* succeed, but fact says otherwise. The patient it is true, may remonstrate, nay, at first determine not to pay—but the legal functionary is immediately employed, and the sufferer not likely to appear in a court of law on a matter to which a degree of ridicule is ridiculously attached, pays the money and keeps his *own* counsel instead of employing one.

The following case for which I pledge my veracity may draw somewhat largely on the credulity of my readers.

A maiden lady, residing at Richmond, applied unknown to her friends, to one of these monarchs of *dental economy*. She made no arrangement as to price—in due time, the *day set* was made, and one for *night* also, and the lady being an invalid, the extortioner's assistant went to Richmond to place them in the mouth. When the feat was accomplished, *five hundred pounds* was demanded as the price of the piece of rascality. For fear of public exposure she paid the money, the worthy's assistant receiving five pounds, as his share of the spoil, as a gratuity.

This very assistant is now practicing on his own account at the west end of London, and no doubt will peruse this article with some degree of curiosity and satisfaction. Whether he fleeces his own patients in the same manner, I will not venture to say, but the above anecdote comes from *his own mouth*. Other cases occur in which gilded brass, or pinchback, is employed in place of gold. Teeth are pivoted with iron, any thing sufficiently durable to last till the patient has paid the fee and got outside the door.

There would be no difficulty in furnishing cases of this kind ad infinitum, and that too among the higher classes of society, who, however, generally speaking, having been foolish enough to employ these cheap dentists have sufficient prudence to keep their own counsel, and not render themselves victims to ridicule as well as fraud. Do not these cases show the degraded state of the dental profession in England, and yet no sooner does any one attempt to expose them than he is *mirabile dictu*, by the respectable portion of the profession of a wish to obtain notoriety. They not having the common sense or common honesty to place themselves in the same position, but contented to remain tame and indolent spectators of a species of rascality, that reflects disgrace on both their profession and themselves. Surely, men who tacitly countenance malpractices of this kind, are little less culpable than those who practice them, more particularly, as they might, by firmness and decision, at once put the public on the irguard, and place the perpetrators *hors de combat.* From what this apathy arises I will not pretend to say. I cannot suppose it possible that they are all influenced by mean and contemptible feelings of *jealousy* that prevents their acting in concert, and can only imagine that they are blessed or cursed with an unenviable portion of lethargy, and care for nothing that does not effect their pecuniary interest. R.

### ARTICLE X.

*Cases of Cancrum Oris.* By J. L. Levison, of Brighton, Eng.

